# Getting ready for the European Health Data Space (EHDS): IDERHA's plan to align with the latest EHDS requirements for the secondary use of health data

**DOI:** 10.12688/openreseurope.18179.1

**Published:** 2024-07-30

**Authors:** Rada Hussein, Irina Balaur, Anja Burmann, Hanna Ćwiek-Kupczyńska, Yojana Gadiya, Soumyabrata Ghosh, Prabath Jayathissa, Florian Katsch, Andreas Kremer, Jaakko Lähteenmäki, Zhaoling Meng, Kathrin Morasek, Rebecca C. Rancourt, Venkata Satagopam, Stefan Sauermann, Simon Scheider, Tanja Stamm, Christian Muehlendyck, Philip Gribbon

**Affiliations:** 1 Ludwig Boltzmann Institute for Digital Health and Prevention, Salzburg, Austria; 2 Luxembourg Centre for Systems Biology, University of Luxembourg, Luxembourg, Luxembourg; 3 Fraunhofer Institute for Software and Systems Engineering, Dortmund, Germany; 4 Discovery Research ScreeningPort, Fraunhofer Institute for Translational Medicine and Pharmacology, Hamburg, Germany; 5 Fraunhofer Cluster of Excellence for Immune-Mediated Diseases (CIMD), Frankfurt, Germany; 6 Bonn-Aachen International Center for Information Technology, University of Bonn, Bonn, Germany; 7 Institute of Medical Information Management, Center for Medical Data Science, Medical University of Vienna, Vienna, Austria; 8 Institute of Outcomes Research, Center for Medical Data Science, Medical University of Vienna, Vienna, Austria; 9 ITTM S.A, Luxembourg, Luxembourg; 10 VTT Technical Research Centre of Finland Ltd, Espoo, Finland; 11 Clinical Modeling and Evidence Integration, Sanofi, Cambridge, MA, USA; 12 Ludwig Boltzmann Institute for Arthritis and Rehabilitation, Vienna, Austria; 13 Medical School Berlin, Berlin, Berlin, Germany; 14 Faculty Life Science Engineering, FH Technikum Wien, Vienna, Austria; 15 Chair for Industrial Information Management, TU Dortmund, Dortmund, Germany; 16 Johnson & Johnson Medical GmbH, Norderstedt, Germany

**Keywords:** Artificial Intelligence; European health data space; cancer, digital health; healthcare standards; interoperability; secondary use of data.

## Abstract

**Objective:**

The European Health Data Space (EHDS) shapes the digital transformation of healthcare in Europe. The EHDS regulation will also accelerate the use of health data for research, innovation, policy-making, and regulatory activities for secondary use of data (known as EHDS2). The Integration of heterogeneous Data and Evidence towards Regulatory and HTA Acceptance (IDERHA) project builds one of the first pan-European health data spaces in alignment with the EHDS2 requirements, addressing lung cancer as a pilot.

**Methods:**

In this study, we conducted a comprehensive review of the EHDS regulation, technical requirements for EHDS2, and related projects. We also explored the results of the Joint Action Towards the European Health Data Space (TEHDAS) to identify the framework of IDERHA’s alignment with EHDS2. We also conducted an internal webinar and an external workshop with EHDS experts to share expertise on the EHDS requirements and challenges.

**Results:**

We identified the lessons learned from the existing projects and the minimum-set of requirements for aligning IDERHA infrastructure with EHDS2, including user journey, concepts, terminologies, and standards. The IDERHA framework (i.e., platform architecture, standardization approaches, documentation, etc.) is being developed accordingly.

**Discussion:**

The IDERHA's alignment plan with EHDS2 necessitates the implementation of three categories of standardization for: data discoverability: Data Catalog Vocabulary (DCAT-AP), enabling semantics interoperability: Observational Medical Outcomes Partnership (OMOP), and health data exchange (DICOM and FHIR). The main challenge is that some standards are still being refined, e.g., the extension of the DCAT-AP (HealthDCAT-AP). Additionally, extensions to the Observational Health Data Sciences and Informatics (OHDSI) OMOP Common Data Model (CDM) to represent the patient-generated health data are still needed. Finally, proper mapping between standards (FHIR/OMOP) is a prerequisite for proper data exchange.

**Conclusions:**

The IDERHA's plan and our collaboration with other EHDS initiatives/projects are critical in advancing the implementation of EHDS2.

## Abbreviations

AI, Artificial Intelligence; CDM, Common Data Model; DCAT, Data Catalog Vocabulary; DCAT-AP, DCAT Application profile for data portals in Europe; DICOM, Digital Imaging and Communications in Medicine; EC, European Commission; EFMI, European Federation for Medical Informatics; EHDS, European Health Data Space; EHR, Electronic Health Record; EMA, European Medicines Agency; EOSC, European Open Science Cloud; FAIR, Findability, Accessibility, Interoperability, and Reusability; FHIR, Fast Healthcare Interoperability Resource; FML, Federated Machine Learning; HHR, Holistic Health Records; HL7, Health Level 7; HTA, Health Technology Assessment; EU, European Union; GDPR, General Data Protection Regulation; IDERHA, Integration of heterogenous Data and Evidence towards Regulatory & HTA Acceptance; JA, Joint Action; LC, Lung Cancer; OHDSI, Observational Health Data Sciences and Informatics; OMOP, Observational Medical Outcomes Partnership; PGHD, Patient-Generated Health Data; RWD, Real-World Data; RWE, Real-World Evidence; TEHDAS, Towards the European Health Data Space; WHO, World Health Organization.

## The IDERHA project

The Integration of heterogeneous Data and Evidence towards Regulatory & HTA Acceptance (IDERHA) project aims to develop one of the first pan-European health data spaces (URL: https://www.iderha.org), and as such, necessitates an adequate adoption of the European Health Data Space (EHDS) principles ^1^. This project has a focus on Lung Cancer (LC), to provide an example of integration and analysis of health data across sectors and along the continuum of care for clinical or medical research questions. There is also an underlying aim to accelerate policy development by building consensus recommendations. These recommendations would further enable the use of heterogeneous health data for product research and development, and are focused on needs of the regulatory and Health Technology Assessment (HTA) community ^2^. IDERHA has selected four use cases in LC to demonstrate the value of health data integration through developing Artificial Intelligence (AI) and Machine Learning (ML) tools in a federated data environment ^3^, and personalized remote monitoring applications for, 1) risk profiling, 2) diagnosis, 3) prognosis, and 4) well-being and patient engagement using device technology/ application in an at-home environment.

We target both institutional and individual data providers. Among the IDERHA consortium partners and within their wider networks, we identified 26 potential institutional data access providers (e.g., institutions, services, repositories) for LC data.

**Figure 1.**
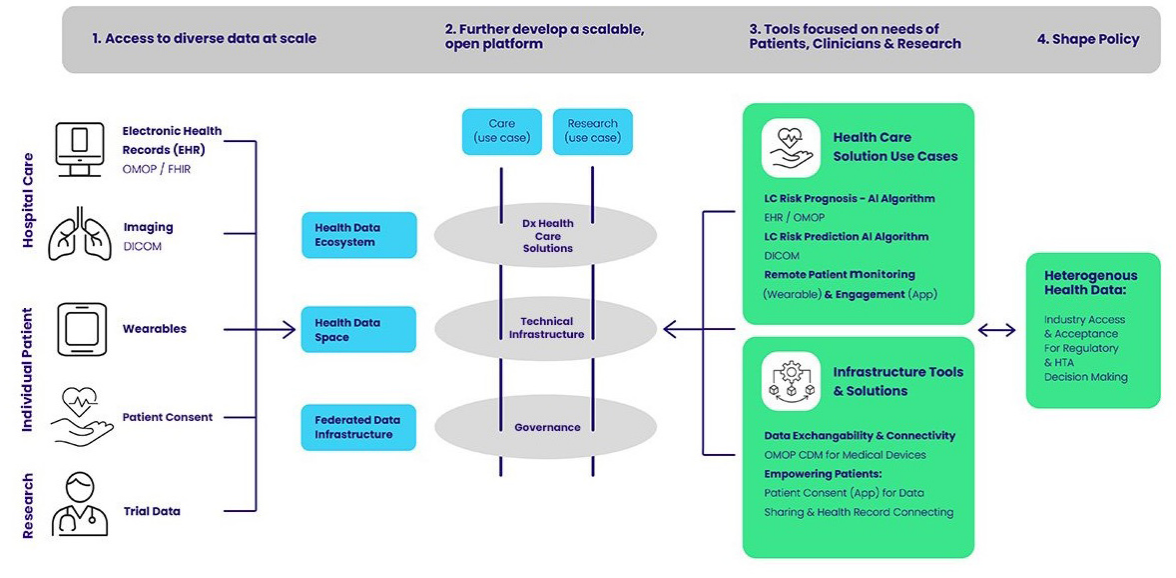
IDERHA Landscape.

From a technical perspective, the IDERHA data space specifies a federated data infrastructure with participatory governance that keeps decision rights distributed among federated parties ^4^. It thus makes health datasets from Data Providers accessible for analysis, including via sophisticated Federated Machine Learning (FML) algorithms ^5^, while ensuring both organizationally (e.g. Data Access Committee (DACs)) and technically (e.g., standardized data policies) enforced access controls. Processing operations of the FML framework are executed at the federated endpoints of the network (i.e., directly at data providers’ sites); subsequently, the partial results of the computations are aggregated at a central node. Thus, the IDERHA infrastructure will facilitate centralized discovery and utilization of federated data resources (i.e., stored, managed, and controlled by the data providers at their facilities) for the evaluation of personal data with privacy-preserving and distributed analytics (see Figure 1). To achieve that, IDERHA aims to connect multiple public and private data sources that aggregate health-related data for secondary use and that cover various aspects of: Electronic Health Records (EHR), clinical images, Patient-Generated Health Data (PGHD), Patient Reported Outcome Measures (PROMs), Patient-Reported Experiences Measures (PREMs), environmental and socioeconomic data.

PROMs for symptom monitoring in cancer provide an evidence-based method for recognizing symptoms, providing clinicians with valuable information, and improving clinical management ^6^. Furthermore, systematically capturing and evaluating patients' perspectives can enhance both their healthcare experience and outcomes. PROMs record symptoms, health-related quality of life, and functional status and refer to standardized questionnaires that are answered directly by the patients. PREMs, on the other hand, focus on the human aspects of the care process ^7^.

The IDERHA project aims to make these heterogeneous health datasets discoverable and utilizable for secondary use in research, innovation, public health, policymaking, regulatory activities, and personalized medicine, by:

- Aligning with the EHDS principles of the secondary use of data ^8^.
- Adopting the principles of Findability, Accessibility, Interoperability, and Reusability (FAIR) ^9^ for both IDERHA data and infrastructure. A metadata catalogue based on FAIR principles ^10^ will support effective data discovery and matchmaking, as well as access to algorithms used in IDERHA. The IDERHA platform will implement a core layer of appropriate authentication and authorization services to manage secure data access.
- Supporting healthcare data standards and models for interoperability ^11^, mainly, Health Level 7 (HL7®) Fast Healthcare Interoperability Resources (FHIR®), Open Health Data Science and Informatics (OHDSI), Observational Medical Outcomes Partnership (OMOP)-Common Data Model (CDM), and Digital Imaging and Communications in Medicine (DICOM®).
- Establishing an IDERHA data quality framework, in which specific requirements and assessment methods will be defined from the data users' perspective ^12^, especially when using Real-World Data (RWD) for decision-making and Real-World Evidence (RWE) ^13^.
- The benefits generated through the execution of use cases on the IDERHA platform will be assessed by a Clinical Advisory Board drawn from key stakeholders, including clinicians and RWE/RWD experts.

This article describes our approach for aligning IDERHA with EHDS2 requirements, highlighting the alignment framework, landscape of existing projects and interoperability standards, lessons learned, and next steps.

## The European Health Data Space (EHDS) regulation

On the 24th of April 2024, the European Parliament adopted the EHDS regulation to build a health-specific ecosystem comprised of rules, common standards and practices, infrastructures, and a governance framework ^8, 14^. The Council will formally adopt the EHDS regulation, which is expected to be published in the Official Journal in autumn. It will then become applicable in different stages according to the use case and data type.

EHDS aims to empower individuals to access and control their health data across the European Union (EU) for 1) the primary use of data (EHDS1) (MyHealth@EU), for healthcare delivery and decision making ^15^ and 2) secondary use of data (EHDS2) (HealthData@EU) for research, innovation, policy-making and regulatory activities ^16^.

In EHDS1, the EU member states will ensure that patient summaries, ePrescriptions, images and image reports, laboratory results, discharge reports among others will be exchanged in a common European format within the cross-border digital infrastructure (MyHealth@EU) ^15^. To ensure that citizens' rights are safeguarded, all member states will appoint digital health authorities that will participate in MyHealth@EU.

**Figure 2.**
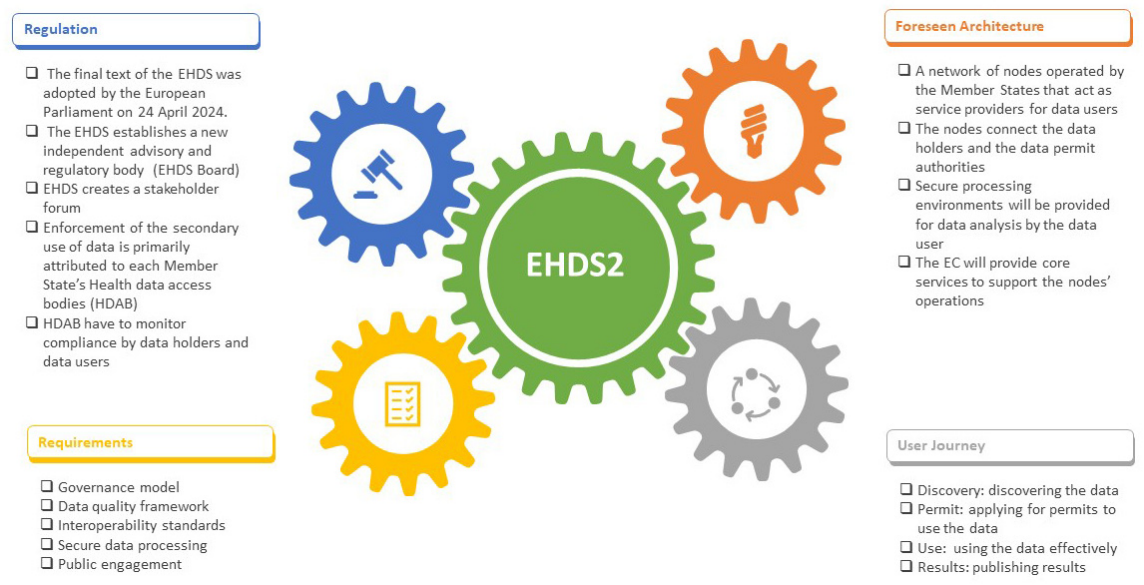
EHDS regulation and EHDS2 foreseen architecture, user journey, and requirements.

On the other hand, in EHDS2 (see Figure 2), each member state will set up a health data access body that gives permits to access data by researchers, companies, or institutions using a decentralized EU-infrastructure (HealthData@EU), which will be set up to support cross-border projects ^16^. The European principles for the secondary use of health data are provided by the TEHDAS Joint Action (JA) (URL: https://tehdas.eu/) and are being adopted by the HealthData@EU pilot project (URL: https://ehds2pilot.eu/).

Because building trust is the main enabler for the success of the EHDS, the EHDS regulation is built further on the General Data Protection Regulation (GDPR), AI Act, the Data Governance Act, the Data Act, and Network and Information Systems (NIS2) Directive ^14^. The EHDS legislation aims to facilitate the sharing of data and leverage opportunities for innovation and still acknowledge data protection and security ^17^. It also requires implementation approaches like IDERHA to overcome organizational and data silos, especially, the EHDS does not provide technical implementation details. Therefore, aligning the IDERHA data space with the EHDS2 principles and the technical requirements provided by the TEHDAS JA is a cornerstone for IDERHA’s synchronization with EHDS and future sustainability.

## The IDERHA’s alignment framework with EHDS2

At first, we conducted a comprehensive review of the EHDS regulation, technical requirements for EHDS2, and related projects that were launched with the EHDS proposal in May 2022. The authors searched PubMed, the European Commission portals, and Google using combinations of terms such as “European Health Data Space,” “secondary use of data” "EHDS", "projects" “infrastructure,” “regulations,” and “standards”. We also used the terms “AI” and “cancer” to search for the main EU-funded projects using AI in cancer since 2020. The first search was conducted in June 2023 and the last search was in January 2024. During the IDERHA internal meetings in November and December 2023, the authors identified 36 projects and categorized them into EHDS1 and EHDS2 projects, personal platforms, cancer projects using AI, and other EHDS supporting projects. We also investigated the deliverables of these projects to identify the lessons learned and the technical approaches that can be adopted during the IDERHA implementation. As the EHDS regulation and related projects are still evolving, we did not apply any exclusion criteria in the planning phase.

The final list of the categorized projects was reviewed by several experts from the IDERHA partners and networks. We also conducted a webinar with EHDS experts in October 2023 to involve their insight and recommendations into the plan. Furthermore, we conducted a workshop with representatives and experts from all IDERHA work-packages in November 2023 to discuss the mapping process of IDERHA architecture with the TEHDAS results and deliverables in terms of concepts, standards, and user journey.

**Figure 3.**
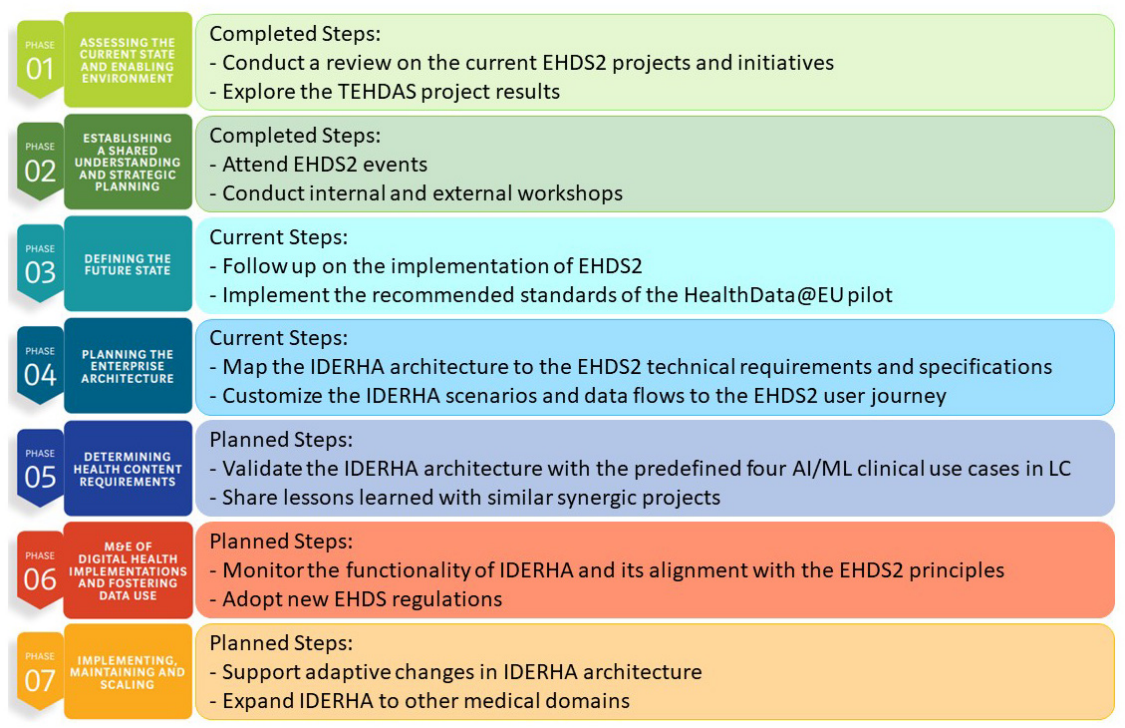
IDERHA's alignment plan with EHDS2 requirements, adapted from the WHO planning tool ^18^.

Finally, we adopted the World Health Organization (WHO) process of planning ^18^ to create the IDERHA's alignment plan with EHDS2. The process comprises seven phases (see Figure 3), as follows:

- In the first phase, we used the results of the conducted comprehensive review to map the current state and enabling environment for EHDS2 and to explore the current projects and initiatives (see Table 1– Table 6). We adopted the European principles for the secondary use of health data provided by the TEHDAS JA and the HealthData@EU pilot project in identifying the minimum set of alignment requirements as listed in Table 7.
- In the second phase, we established a shared understanding and strategic planning with internal and external experts through conducting and attending several EHDS2 events, e.g., meetings, webinars, workshops, etc. In October 2023, we also organized a workshop with EHDS experts and IDERHA consortium members, where we discussed the potential impact of the EHDS on the implementation of IDERHA. We also identified key areas of common interest and priority topics for the upcoming workshops.
- In phase 3, we explore the future state of the EHDS2 implementation through networking with other thematically aligned projects, e.g., the HealthData@EU pilot, the European Federation for Cancer Images (EUCAIM) (URL: https://www.eibir.org/projects/eucaim/), and the EHDS2 recent implementation projects in 2024: TEHDAS2 JA and the EHDS Data Quality and Utility Label (Quantum) project.
- In phase 4 for planning enterprise architecture, we currently map the EHDS2 specifications and user journey to the IDERHA architecture.
- To realize phase 5 for determining health content requirements, we will validate the IDERHA architecture with the predefined four AI clinical use cases in LC. Moreover, we currently participate in the HSbooster.eu (URL: https://hsbooster.eu/) to get consultation services and the OMOP and HL7/FHIR standards for the PGHD and reported health outcomes. Additionally, we created synergies with similar projects for sharing lessons learned and extending expertise in PGHD collection ^19^, integration with EHR ^20^ and establishing need for standardization, for example, the Holistic Health Record approach ^21^ adopted by the iHelp project (URL: https://ihelp-project.eu/).
- After the development of the IDERHA infrastructure, we will start with phase 6 for Monitoring and Evaluation (M&E) and fostering infrastructure use. We will monitor the functionality of the IDERHA data space and its alignment with the EHDS2 principles. We will also aim to ensure the adoption of the EHDS regulation for data access and sharing.
- We aim to provide a scalable open architecture of IDERHA to support both the clinicians’ and researchers’ journey in alignment with the EHDS2 data governance principles. We also plan to build synergies with other EHDS2 projects (fulfilling phase 7) to implement, maintain, and scale IDERHA to other medical domains.

**Table 1.** Main EHDS initiatives and projects: Primary use of data (EHDS1).

Initiative/ Project	Scope/Goal	Standards	Lessons Learned
MyHealth@EU	EC cross-border infrastructure for patient’s data exchange in healthcare delivery	eHealth Digital Service Infrastructure (eHDSI) EEHRxF	ePrescriptions and Patient Summary (long term, medical images, lab results and hospital discharge reports) ^19^
X-eHealth	Accelerating the implementation of the EEHRxF	EEHRxF	Implementation and deployment of EEHRxF services ^20^
XpanDH	Empower individuals and organizations to create, adapt, and explore interoperable digital health solutions	EEHRxF	Successful adoption of the EEHRxF ^21^
Xt-EHR	Joint Action carried out by 25 European countries for laying the groundwork for the improved primary use of electronic health data for EHDS	EEHRxF	The project will develop the implementation guides, technical specifications, and a conformity assessment framework for the adoption of the EEHRxF at the European level

**Table 2.** Main EHDS initiatives and projects: Secondary use of data (EHDS2).

Initiative/Project	Scope/Goal	Standards	Lessons Learned
HealthData@EU pilot	Piloting an infrastructure for the secondary use of health data	Extension of DCAT-AP: HealthDCAT-AP (Expected in early 2024)	Proof-of-concept implementation ^25^, and exploration of the legal landscape ^26^
TEHDAS1	Joint Action carried out by 25 European countries to develop the European principles for the secondary use of health data	Examined the standards for health data discovery, as well as enabling semantic and exchange interoperability	Identification of technical and data governance requirements for EHDS2 alignment ^27^
Genomic Data Infrastructure (GDI)	Enabling access to genomic and related phenotypic and clinical data across Europe.	DCAT-AP	User journey, federated data access scenarios ^28^, connectivity with EHDS and EOSC
EUCAIM	A pan-European digital federated infrastructure of FAIR cancer-related, de-identified, real-world images.	OMOP, FHIR, DICOM, DCAT-AP	Development, benchmarking, testing, and piloting of AI-based technologies for cancer diagnosis and treatment ^29^ and federated data access scenarios ^30^
DARWIN EU	Delivering real-world evidence from across Europe on diseases, populations and the uses and performance of medicines.	OMOP	Federated data analysis network operational, participation in HealthData@EU Pilot, and incorporating RWE in HTA ^31^

**Table 3.** Main EHDS initiatives and projects: Personal platforms.

Initiative/Project	Scope/Goal	Standards	Lessons Learned
Gravitate Health	Foster personal health management and adherence to treatment	FHIR HL7 Vulcan	Personal Health Data Space ^32^
AIDAVA	Automating curation and publishing of Personal Health Data through AI	DCAT-AP, SNOMED, FHIR, LOINC, IPS & EEHRxF	Personal Health Knowledge Graphs (PHKG) ^33^

**Table 4.** Main EHDS initiatives and projects: AI/ML in Cancer.

Initiative/Project	Scope/Goal	Standards	Lessons Learned
ASCAPE	Creating an open AI infrastructure enabling deployment and execution of AI algorithms locally and results to be shared	EN/ISO 13606, SNOMED CT, LOINC, HL7 CDA & FHIR	ML techniques in cancer ^34^, PGHD integration with EHR ^35^
iHelp	Personalized Health Monitoring and Decision Support Based on AI and Holistic Health Records for early identification and mitigation of risks associated with Pancreatic Cancer	FHIR	Collection, integration, and management of health-related data from various sources in standardized Holistic Health Records (HHR) ^36^
OPTIMA	Enable shared decision-making using dynamic computer-interpretable guidelines (CIGs), access to broad data sets, AI algorithms and tools	OMOP, FHIR, DICOM	Federated network of data providers on cancer, computer interpretable guidelines ^37^
UNderstand CANcer (UNCAN.eu)	Creating the UNCAN.eu platform	-	The blueprint for UNCAN.eu proposed to set up a European Federated Cancer ^38^
INCISIVE	A multimodal AI-based toolbox and an interoperable health imaging repository	FHIR, DICOM, SNOMED, LOINC	Tailoring the legal framework, adopting technological solutions for privacy-preserved data collection, integration, and harmonization, and how federated data storage and sharing has been achieved ^39^

**Table 5.** Main EHDS initiatives and projects: EHDS2 supporting and interlinking activities.

Initiative/Project	Scope/Goal	Standards	Lessons Learned
XShare	Enable personal health data sharing through EHRxF.	EHRxF	Personal Health Data Portability, Standard and Policy development
HSBooster.eu	Standardization consultancy service to EU-funded projects	International Organization for Standardization (ISO) standards	Synergies between standardization projects
FHIR for FAIR - FHIR4FAIR	Guidance on how HL7 FHIR can be used for supporting FAIR health data implementation	FHIR	Leveraging FHIR in health data FAIRfication process ^40^
Hospitals On FHIR	Establishing Hospitals on FHIR network in Europe	FHIR	Preparing Hospitals for European Health Data Space by introducing an interoperability capabilities maturity model ^41^
Data Spaces Support Centre (DSSC)	Contribute to the creation of common data spaces	-	Definition of common requirements and best practices on building data spaces ^42^
GAIA-X	Initiative to develop a digital governance that can be applied to any existing cloud/ edge technology stack to obtain transparency, controllability, portability and interoperability across data and services.	-	Framework specification covering compliance, trust, federation and data exchange ^43^
International Data Spaces Association	Initiative to enable secure, sovereign data sharing across companies and industries ensuring self-determined control of data use for data providers	-	Framework specification for data exchange and dataspace interoperability ^44^
FAIRplus	Increase the discovery, accessibility and reusability of data from selected IMI projects, as well as internal data from pharmaceutical industry	Endorsement of domain-specific standards	Assessment of FAIR datasets to ensure community reuse, guidelines and tools on FAIRification, e.g. FAIR cookbook ^45^
European Health Data Evidence Network – EHDEN	Building a large-scale federated network of data sources standardized to a common data model, following the FAIR approach	OMOP	Metadata on data sources in a database catalogue ^46^
Health Outcomes Observatory – H2O	Create ‘health outcomes observatories’ that will amplify the patient voice both in their own healthcare and in healthcare systems more broadly, by establishing a data governance and infrastructure system initially in four European countries	OMOP, FHIR	Network of observatories, standardized core outcome sets for diabetes, inflammatory bowel disease and cancer ^47^
EOSC life	Create an open, digital and collaborative space for biological and medical research with 13 Life Science ‘ESFRI’ research infrastructures	-	Tools and best practices, e.g. Clinical Research Metadata Repository, Ontology Lookup Service ^48^
HealthyCloud	Generating several guidelines, recommendations and specifications that will enable distributed health research across Europe in the form of a Ready-to-implement Roadmap.	-	Strategic agenda towards the European Health Research and Innovation Cloud (HRIC) - proposal for five core services ^49, 50^
Personal Health Train	Giving controlled access to data, while ensuring privacy protection and optimal engagement of individual patients and citizens.	-	Implementation reference for decentral data analysis, FML demonstrated ^51, 52^
GO FAIR	Open ecosystem for collaboration on FAIR data and services organized in implementation networks	Metadata standards per domain	Operational implementation networks, e.g. on personal health train ^53^
HEALTH-X dataLOFT	Implementing legitimized, open, and federated dataLOFT platform and made accessible to citizens	FHIR, SNOMED-CT, IHE, Gaia-X interfaces	Data Space implementation ^54^
InteropEHRate	Enabling patient-centered exchange of health records	FHIR	End-to-end data integration methodology and open protocol specifications ^55^
Sphin-X	Enabling sovereign collaboration with health data from solution provider via healthcare provider to patient	-	End-to-end community and ecosystem building

**Table 6.** Main EHDS initiatives and projects: Ongoing and upcoming EHDS implementation projects.

Initiative/Project	Scope/Goal
TEHDAS2 (May 2024- December 2026)	Supporting the successful realization of the EHDS, where data would be available securely on demand across borders for patient care (primary use) and for secondary purposes such as research, innovation, and policymaking.
QUANTUM (January 2024 - June 2026)	Performing a mapping of existing data quality and utility principles, initiatives, and frameworks (i.e. EMA/ Heads of Medicines Agencies (HMA), TEHDAS, EOSC-LIFE, Health Data Research UK’s data quality and utility framework, and relevant data principles, resources and tools (FAIR, FAIR Cookbook, etc.)
EC and WHO/Europe agreement in December 2023 (Harnessing health data, 4-Year project funded by the EU4HEALTH programme)	The project aims to strengthen health information systems and boost health data governance and interoperability in Europe. The initiative will be driven by the EHDS framework and principles to facilitate the use and reuse of health data within the EU ^56^

**Table 7.** IDERHA alignment with EHDS2 requirements based on the TEHDAS results.

TEHDAS deliverable	Scope	Mapping to IDERHA
Deliverable 5.4: Options for governance models for the European Health Data Space ^58^	Data governance	Understanding the EHDS2 legal interoperability and how it might be used, customized or reflected to the IDERHA data lifecycle and associated data governance models.
Deliverable 7.2: Options for the services and services architecture and infrastructure for secondary use of data in the EHDS ^59^	User journey	Adopting the proposed EHDS2 user journey, concepts, and terminologies into the IDERHA patient/researcher scenarios and overall architecture (see Figure 4).
Deliverable 6.2: Recommendations to enhance interoperability within HealthData@EU ^60^	Standards and interoperability	Considering the recommended standards for data discovery (DCAT-AP), enabling semantic interoperability (OMOP) and data exchange (DICOM and FHIR).
Deliverable 6.3: Recommendations on a Data Quality Framework for the European Health Data Space for secondary use ^61^	Data quality	Following the provided Data Quality Framework in developing IDERHA's data quality approach.
Deliverable 5.3: Guidelines document for multicountry data access applications, including mutual recognition and cross-border applications ^62^	Data access applications	Guiding in developing the IDERHA's data access approaches with the EHDS2 perspective on data access and data permit processes in different national settings.

## The landscape of existing projects and interoperability standards

The conducted comprehensive review explored the current state and enabling environment for EHDS2. The following tables summarize the main projects and initiatives shaping the EHDS, the recommended standards for health data interoperability in these projects, and the lessons learned that are relevant to IDERHA. Table 1 addresses the main existing projects shaping the MyHealth@EU using the European Electronic Health Record Exchange Format (EEHRxF) 

Table 2 mainly addresses the HealthData@EU pilot and TEHDAS1 JA. It also introduces the other projects that support the EHDS2 implementation. The current key standards for EHDS2 are OMOP, FHIR, DICOM, and DCAT-AP.

Table 3 explores the projects that build personal platforms that empower patients to take an active role in managing and sharing his/her health data in alignment with the EHDS2. The main standards used in the personal health data space are FHIR, HL7 Vulcan, DCAT-AP, and others.

Table 4 highlights similar EU-funded projects that utilize AI and ML in the cancer domain. Besides using the OMOP, FHIR, and DICOM standards, the iHelp and ESCAPE projects adopt innovative approaches for personalized healthcare through the integration of personal data with EHR.

Table 5 lists the major projects that support the implementation and standardization of EHDS, in terms of”

- EHDS architecture and main principles, such as GAIA-X, Data Spaces Support Centre – DSSC, etc.
- European research infrastructure, such as, EOSC life, HealthyCloud
- Standardization, such as XShare, HSBooster.eu, EHDEN, Hospitals On FHIR projects, etc.
- Data FAIRfication, such as FAIRplus, GO FAIR, etc.

Finally, Table 6 highlights the recently kicked-off projects concerning the real-world implementation of the EHDS and related technical needs.

### IDERHA’s minimum set of requirements for EHDS2 alignment

The TEHDAS JA developed the European principles for the secondary use of health data with the involvement of 25 countries. The results of TEHDAS are currently adopted to shape the HealthData@EU pilot project, mainly the user journey ^57^. Similarly, we selected the relevant TEHDAS guidelines and recommendations that would be considered in IDERHA (as listed in Table 7).

**Figure 4.**
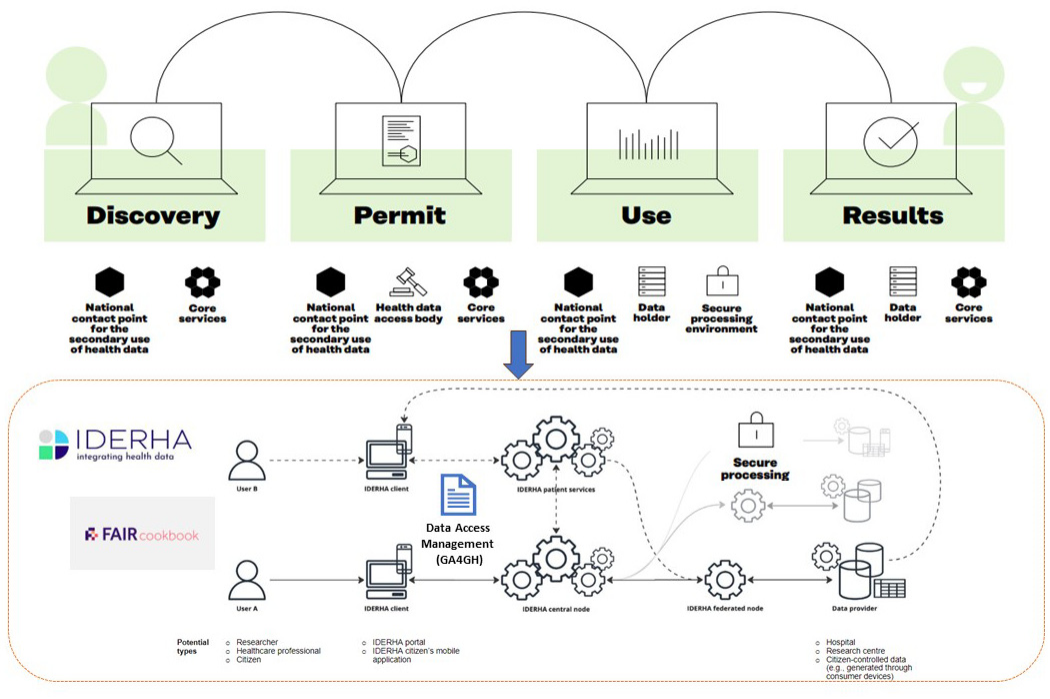
IDERHA infrastructure in alignment with EHDS2 user journey, adapted from 57.

Figure 4 provides an overview of the IDERHA platform, including its potential actors/components and their roles. Two exemplary scenarios/data flows are presented for two types of users: Researcher (federated data analysis use case) and Citizen (own personal health data access). These processes are aligned with the EHDS2 user journey, covering discovery, permit, use, and results processes.

**Figure 5.**
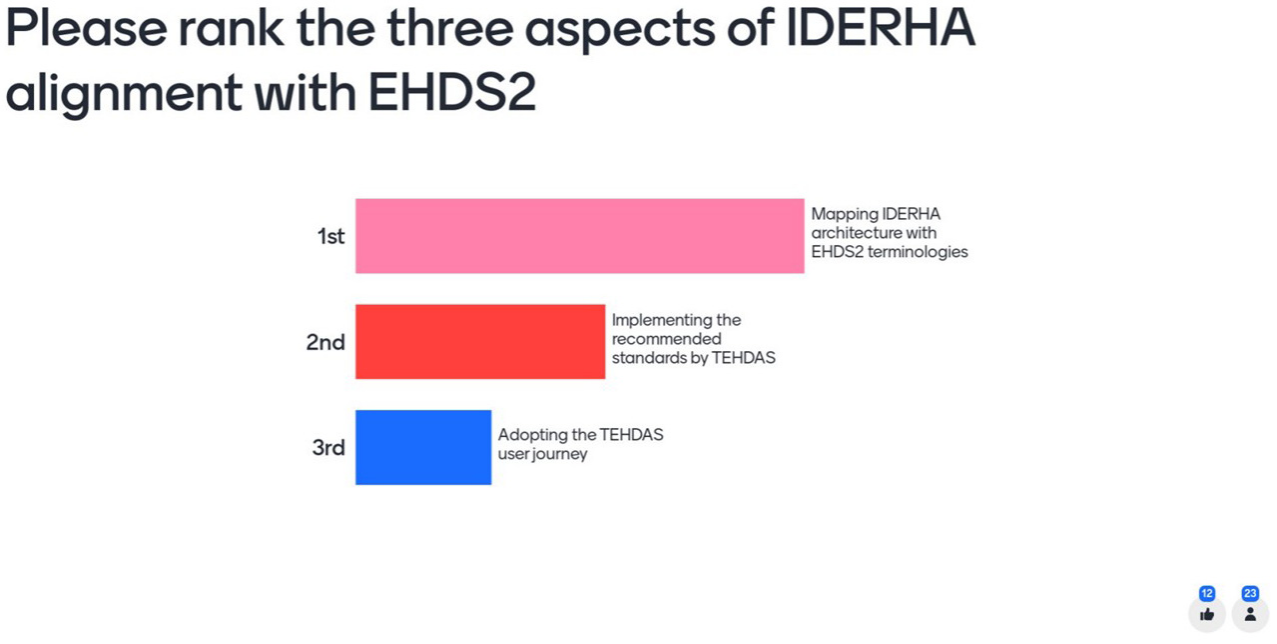
Mentimeter results ranking the main aspects for aligning IDERHA with EHDS2.

Figure 5– Figure 9 and Table 8, Table 9 provide the detailed results of mapping the EHDS2 requirements to IDERHA using the TEHDAS deliverables.

**Figure 6.**
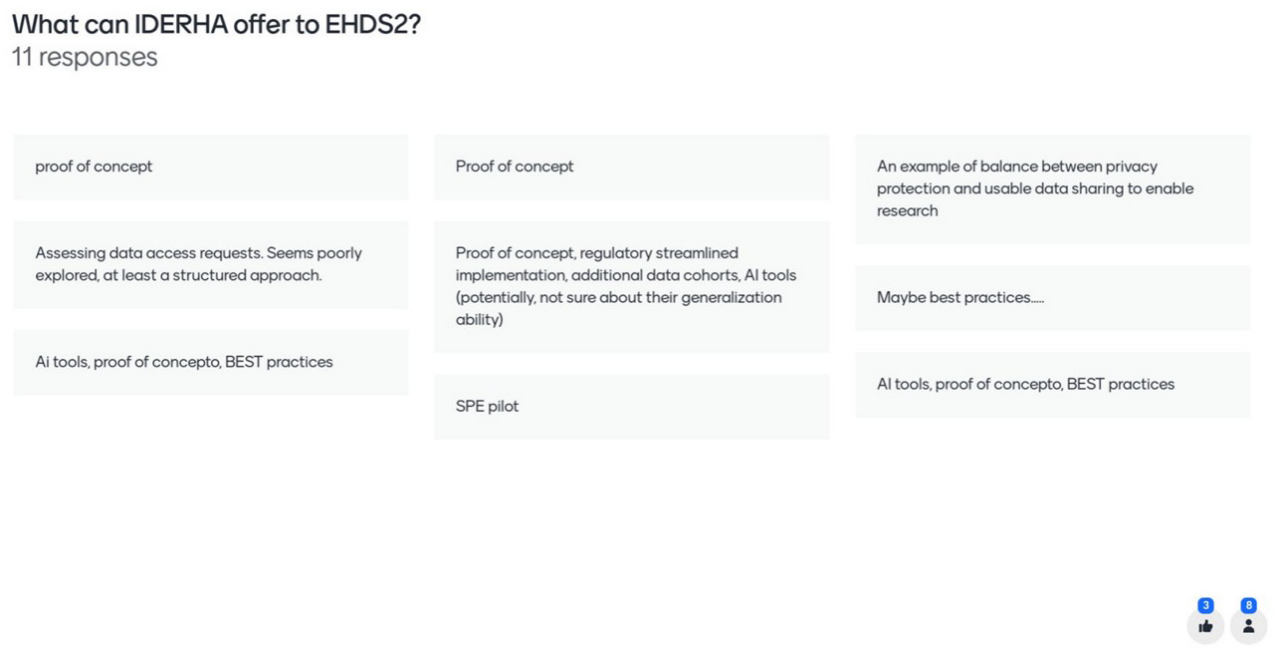
Mentimeter results – how can IDERHA shape the EHDS2 implementation (1/2).

**Figure 7.**
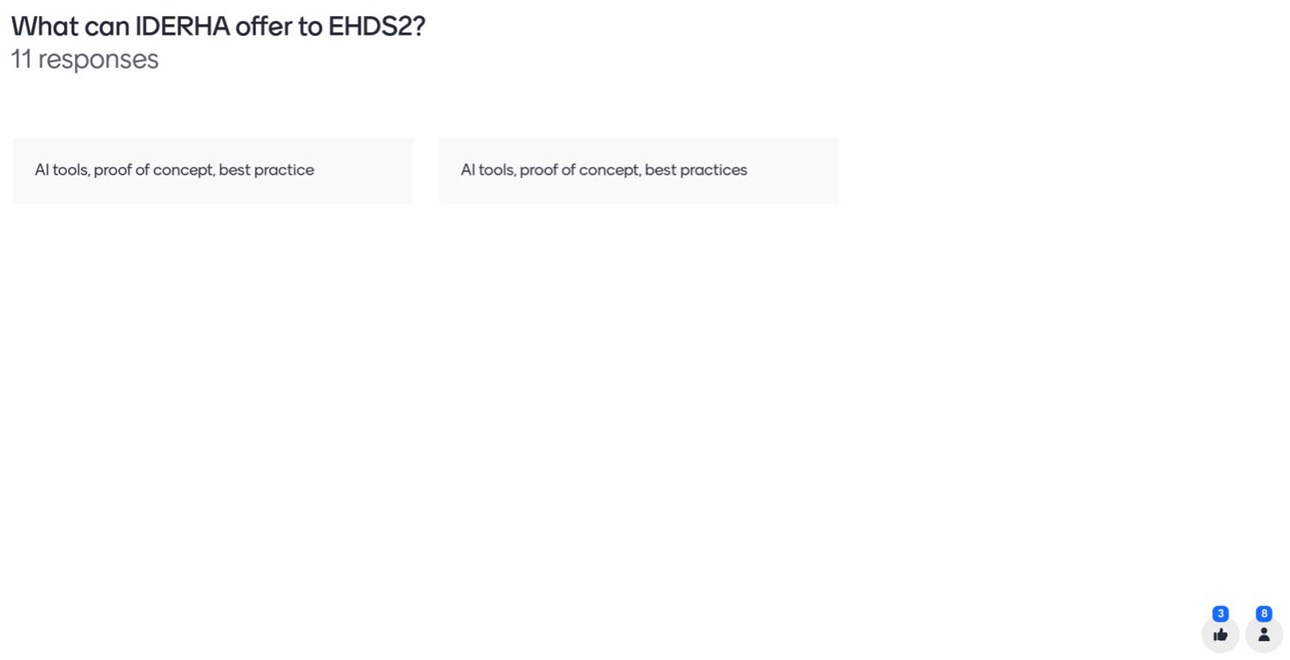
Mentimeter results – how can IDERHA shape the EHDS2 implementation (2/2).

**Figure 8.**
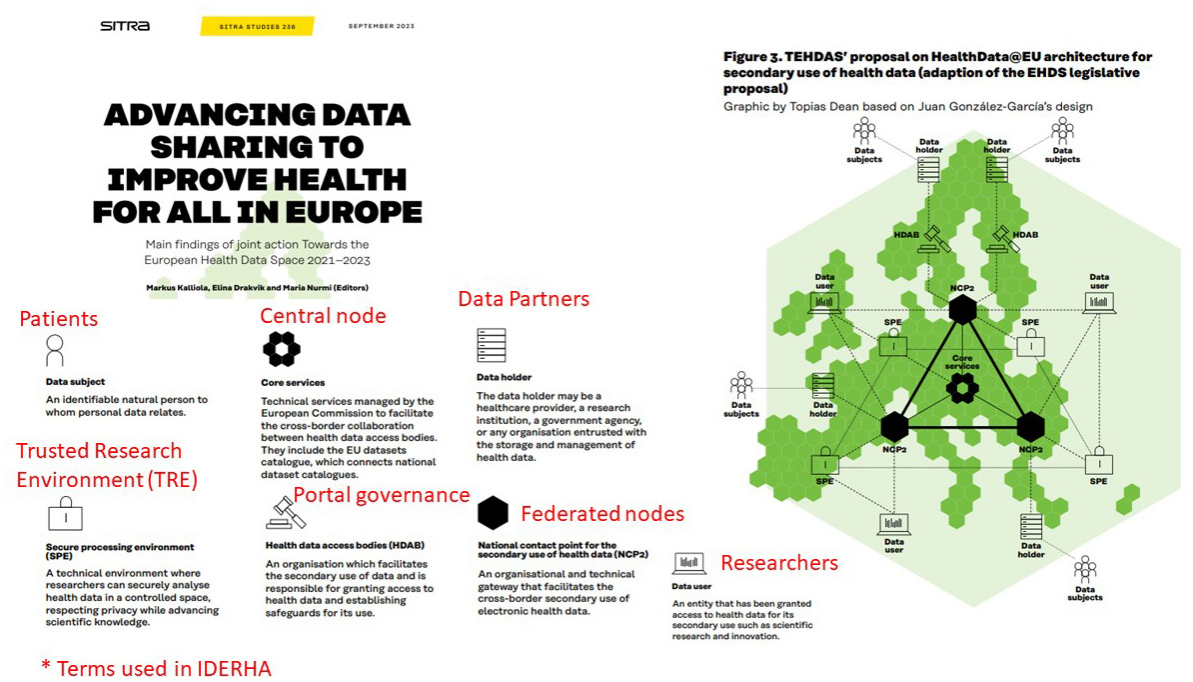
Mapping the EHDS2 terminologies and concepts to IDERHA ^57^.

**Figure 9.**
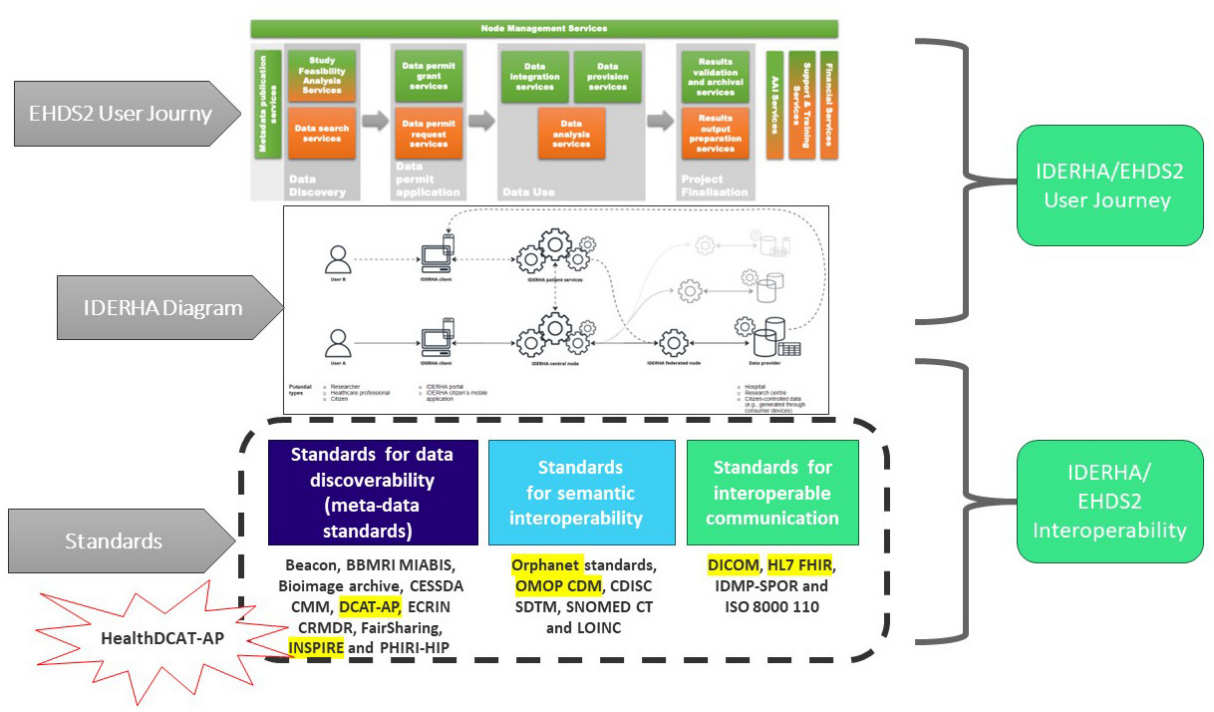
Mapping the TEHDAS user journey and recommended standards to IDERHA ^59^.

**Table 8.** Mapping the EHDS2 terms to IDERHA.

EHDS2 terms	Mapping to IDERHA
**Data User**	Yes
**Data subject**	Yes
**Secure processing environment** **(SPE)**	Planned
**Core services**	Yes
**Health data access bodies (HDAB)**	TBD
**Data holder**	Yes
**National contact point for the** **secondary use of health data (NCP2)**	TBD

**Table 9.** Mapping the EHDS2 recommended standards to IDERHA.

EHDS2 Standards	Used Standards in IDERHA
Data Discoverability	TBD
Enabling semantic Interoperability for the secondary use of health data	OMOP CDM

### The IDERHA data space

IDERHA adopts the principles of the EHDS2, and it is oriented towards the Gaia-X principles (i.e., decentralization, data sovereignty, federation) and developments of important European initiatives (see Table 5). Thus, the IDERHA architecture essentially relies on two core processes: (1) Data Access Request and (2) FML Execution. The federated architecture of IDERHA along with the associated Secure Processing Environments (SPEs), alongside data FAIRification meets the currently proposed EHDS2 user journey and service requirements. In addition, the data governance model of IDERHA is realized through synchronization and linking between the Data Management Plan (DMP), the Data Protection Impact Assessment (DPIA), and the Data Sharing Agreements (DSA) with data partners.

We plan to implement the recommended standards for data discovery (DCAT-AP), enabling semantic interoperability (OMOP) and data exchange (DICOM and FHIR). We will also investigate the possibility of implementing the extension of the DCAT-AP for health (HealthDCAT-AP) that is being developed by the HealthData@EU pilot project. Furthermore, we build synergy with similar projects through the HSbooster.eu activities and the European Health Data Evidence Network (EHDEN) to share expertise in healthcare standards and interoperability, especially proposing extensions to standards development organizations for PGHD and the need for addressing the mapping challenges between the different standards, for example, mapping OMOP and HL7 FHIR ^46^.

As the EHDS infrastructure and requirements are still evolving ^63^, we will continue to share the lessons learned among the related projects, e.g., EUCAIM, GDI, HealthData@EU pilot, TEHDAS2, the European Federated Cancer Research Data Hub, and others (see Table 2).

### Recommendations to International and European Organizations

For efficient implementation of the EHDS2 ecosystem, the authors recommend establishing communication channels and foster networking between all stakeholders, for instance:

- The EC can promote more collaboration and build synergy among EHDS projects, for example the HSbooster.eu pilot provides a framework for gathering forces for standardization. This model can be also extended to build the EHDS2 community in addition to the EC planned activities for capacity building.
- The WHO/Europe can provide designated EHDS alignment toolkits, M&E framework, and an EHDS atlas for locating the national health data access bodies, data registries, projects, etc. matching with the WHO/Europe digital health roadmap action plan for the WHO European Region 2023–2030 ^64^. This is in addition to the EU and WHO/Europe and the EC new partnership to strengthen health information systems and boost health data governance and interoperability in the WHO European Region.
- The European Federation for Medical Informatics (EFMI) can provide expertise for modeling and building an interoperability as a service layer to facilitate the connectivity of data holders to the EHDS infrastructure.

## The way ahead

The IDERHA project aims to provide a disease and use case-agnostic framework for federated access and processing of anonymized and pseudonymized health data, ensuring data protection and sovereignty through state-of-the-art privacy-preserving technologies. This work describes our plan to align IDERHA with the EHDS2 requirements, including, user journey, services and architecture, and standards. This described framework for aligning IDERHA with EHDS2 requirements can be used as a template for similar and upcoming projects.

The next step is to implement this plan and monitor the outcomes. Concurrently, we will follow up the development of the HealthData@EU to consider the new recommendations for proper implementation of the regulation and better health data interoperability. In addition, we establish a dialogue with similar projects and related organizations to share expertise in implementing the EHDS infrastructure. In this way, IDERHA will actively participate in shaping EHDS2 as one of the first pan-European initiatives.

## Ethics and consent

Ethics and consent were not required.

## Data Availability

There are no new data associated with this article.
