# Supplementary material for: Getting ready for the European Health Data Space (EHDS): IDERHA's plan to align with the latest EHDS requirements for the secondary use of health data
Source: Open Res Eur. 2024 Jul 30;4:160. [Version 1] doi: 10.12688/openreseurope.18179.1 (PMC11342032; doi:10.12688/openreseurope.18179.1)
Supplement: Peer review history

# Reviewer report: Reviewer response for version 1

**DOI:** 10.21956/openreseurope.19648.r42746

**Published:** 2024-09-11

**Authors:** Subhashis Das

**Affiliations:** 1 Universidad de Salamanca, Dublin, Castile and León, Spain

This paper presents the IDERHA project in the context of implementing a pan-European health data space according to the EHDS regulation and, more particularly, the secondary use of health data for research and innovation. The project uses lung cancer as a pilot for the integration of heterogeneous health data, with specific emphasis on interoperability, standardization, and AI-driven analysis within a federated infrastructure. The authors review EHDS regulations and existing projects, identify challenges, and propose solutions for aligning the IDERHA platform with EHDS2, particularly in terms of data standards, privacy, and interoperability. Table 8. Mapping the EHDS2 terms to IDERHA. Table 9. Mapping the EHDS2 recommended standards to IDERHA is well explained. The author may have a look at some **Deliverables from Interoperate project.** https://www.interopehrate.eu/resources/#pubs

Is the study design appropriate and does the work have academic merit?

Yes

Is the work clearly and accurately presented and does it cite the current literature?

Yes

If applicable, is the statistical analysis and its interpretation appropriate?

Not applicable

Are all the source data underlying the results available to ensure full reproducibility?

No source data required

Are the conclusions drawn adequately supported by the results?

Yes

Are sufficient details of methods and analysis provided to allow replication by others?

Yes

Reviewer Expertise:

Health Informatics, Healthcare standards, EHR, Data integration, Ontology, Knowledge Graph

I confirm that I have read this submission and believe that I have an appropriate level of expertise to confirm that it is of an acceptable scientific standard.

# Reviewer report: Reviewer response for version 1

**DOI:** 10.21956/openreseurope.19648.r42743

**Published:** 2024-08-22

**Authors:** Filipe Andrade Bernardi

**Affiliations:** 1 University of São Paulo, São Paulo, Brazil

**Summary :**

 This paper presents the IDERHA project in the context of implementing a pan-European health data space according to the EHDS regulation and, more particularly, the secondary use of health data for research and innovation. The project uses lung cancer as a pilot for the integration of heterogeneous health data, with specific emphasis on interoperability, standardization, and AI-driven analysis within a federated infrastructure. The authors review EHDS regulations and existing projects, identify challenges, and propose solutions for aligning the IDERHA platform with EHDS2, particularly in terms of data standards, privacy, and interoperability.

**Assessment**

Clarity and Accuracy (Yes):

 The article is clear and well-organized. The concepts are explained thoroughly, and it cites current, relevant literature. No major changes are needed.

Study Design and Academic Merit (Yes):

 The study is well-designed and addresses an important issue in health data management. It uses a real-world case (lung cancer) to show how the system can work, which adds practical value. No revisions are needed here.

Methods and Analysis (Partly):

 The article lacks detailed technical information about how the project is being implemented. For instance, it should better explain how specific data standards (OMOP, FHIR, DICOM) are applied and how data privacy is maintained.

**Recommendation** : Add more specific details about the technical steps used in the project so others could replicate it.

Statistical Analysis (Not Applicable):

 This article doesn’t involve statistical analysis, so this doesn’t apply.

Source Data (No Source Data Required):

 The article doesn’t use new data or experiments, so no source data is needed.

Support of Conclusions (Yes):

 The conclusions are well-supported by the content of the article. They align with the analysis and offer practical insights into the challenges and solutions for aligning with EHDS2.

**Key Points for Improvement:**

 Add Technical Details: To make the work easier to replicate, include more details about how the interoperability standards and privacy measures are implemented.

Is the study design appropriate and does the work have academic merit?

Yes

Is the work clearly and accurately presented and does it cite the current literature?

Yes

If applicable, is the statistical analysis and its interpretation appropriate?

Not applicable

Are all the source data underlying the results available to ensure full reproducibility?

No source data required

Are the conclusions drawn adequately supported by the results?

Yes

Are sufficient details of methods and analysis provided to allow replication by others?

Partly

Reviewer Expertise:

Health Data Interoperability; Health Data Privacy and Security

I confirm that I have read this submission and believe that I have an appropriate level of expertise to confirm that it is of an acceptable scientific standard.

## References

[1] : Privacy‐preserving data mining and machine learning in healthcare: Applications, challenges, and solutions. *WIREs Data Mining and Knowledge Discovery* .2023;13(2) : 10.1002/widm.1490 10.1002/widm.1490

[2] : Data Quality in Health Research: Integrative Literature Review. *J Med Internet Res* .2023;25: 10.2196/41446 e41446 10.2196/41446 37906223 PMC10646672

# Response

**Published:** 2024-08-22

**Authors:** Rada Hussein

**Affiliations:** Ludwig Boltzmann Institute for Digital Health and Prevention, Salzburg, Salzburg, Austria

Dear Prof. Filipe Andrade Bernardi, Thank you for your valuable feedback. We will add more technical details upon finalizing the next phases of the plan in the new version. Best Regards, Rada

# Reviewer report: Reviewer response for version 1

**DOI:** 10.21956/openreseurope.19648.r42747

**Published:** 2024-08-22

**Authors:** Snezana Savoska

**Affiliations:** 1 University St. Kliment Ohridski, Bitola, North Macedonia

This is an interesting paper for IDERHA project, with aims to develop one of the first pan-European health data spaces (URL: https://www.iderha.org).

 In my opinion, the project aims, methods and intention are well expressed in the paper and it is a good and comprehensive narration for such an integral and complex project with presentations in visual format of the processes, environment and workflows.

 For me, it is a very important project that aims to connect the research from others EU project in the area, creating EU health data spaces with adoption of the European Health Data Space (EHDS) principles and standards.

Is the study design appropriate and does the work have academic merit?

Yes

Is the work clearly and accurately presented and does it cite the current literature?

Yes

If applicable, is the statistical analysis and its interpretation appropriate?

Yes

Are all the source data underlying the results available to ensure full reproducibility?

Yes

Are the conclusions drawn adequately supported by the results?

Yes

Are sufficient details of methods and analysis provided to allow replication by others?

Yes

Reviewer Expertise:

ICT, e-Health, IoT, Information systems, e-Health and Electronic Health Record, Patient Health record, Hospital Information systems

I confirm that I have read this submission and believe that I have an appropriate level of expertise to confirm that it is of an acceptable scientific standard.

# Response

**Published:** 2024-08-22

**Authors:** Rada Hussein

**Affiliations:** Ludwig Boltzmann Institute for Digital Health and Prevention, Salzburg, Salzburg, Austria

Dear Prof Snezana Savoska, Thank you for your positive comments. Best Regards, Rada

# Reviewer report: Reviewer response for version 1

**DOI:** 10.21956/openreseurope.19648.r42751

**Published:** 2024-08-16

**Authors:** Gunnar Piho

**Affiliations:** 1 Tallinn University of Technology, Tallinn, Estonia

The article titled "Getting ready for the European Health Data Space (EHDS): IDERHA's plan to align with the latest EHDS requirements for the secondary use of health data" by Hussein et al. offers a comprehensive examination of the IDERHA project, a pan-European initiative designed to align with the European Health Data Space (EHDS) regulations. The article primarily focuses on the secondary use of health data, with lung cancer as the pilot project.

 The authors systematically discuss the regulatory framework established by the EHDS, highlighting the importance of standardisation and interoperability in health data management across Europe. The article underscores the challenges and opportunities the EHDS regulation presents, particularly the necessity for integrating heterogeneous data sources and implementing robust data governance frameworks.

 A notable strength of the article lies in its thorough review of existing projects and standards that inform the development of the IDERHA platform. The authors provide a detailed overview of the technical requirements, including adopting standards such as OMOP, FHIR, and DICOM, while addressing the challenges related to data discoverability and semantic interoperability.

 The article employs a rigorous methodological approach, incorporating a combination of literature review, expert consultations, and workshops. This methodology enables the authors to present a well-rounded perspective on aligning IDERHA with EHDS requirements. Furthermore, the article offers valuable insights into lessons learned from other projects, which play a crucial role in shaping IDERHA’s strategic alignment with EHDS2.

 However, the article has room for improvement, particularly in terms of detail and readability. At times, the content can be challenging to comprehend. I believe the following corrections and improvements could enhance the article:

1. All references and corresponding links need to be carefully checked. At least references 32 and 33 in Table 3, along with their respective links, appear incorrect and do not lead to the intended sources. While I did not check all references, the authors should undertake this task thoroughly.
2. The necessity of Figures 5, 6, and 7 is unclear. In my view, they are redundant. These figures are not adequately explained in the text, and I believe their content could be more effectively conveyed through regular text rather than as figures.
3. The content and explanations of Figures 1-3 are well-constructed and comprehensible, as are the explanations for Tables 1-6.
4. On page 10, towards the end, the phrase "The following tables summarize ..." is somewhat confusing. I suggest revising it to "Tables xx - yy summarise ... ".
5. The lower part of Figure 4 contains text that is too small. This figure could benefit from a more detailed explanation, clarifying what is depicted. If there are two types of users, it would be helpful to explain how these users interact with the data shown in the figure.
6. The content of Figure 8 could be more effectively presented as a table. Additionally, it would be beneficial to explain why there has been a shift in terminology.
7. Figure 9 is mainly illegible. Furthermore, this figure needs a textual explanation, which I either missed or overlooked.
8. I find Tables 8 and 9 to be superfluous.
9. It might be helpful to provide more detailed explanations regarding the organised workshop and the profiles of the experts who participated to lend greater credibility to their assessments. As it stands, this aspect feels somewhat underdeveloped.
10. I also recommend elaborating on the contribution and significance of the IDERHA project. A robust framework could be presented, emphasising the project's objectives (as outlined on page 4), the path to achieving these objectives (Figure 3), and in the analysis and discussion sections, summarising why this particular approach was chosen and what it is expected to achieve.

In conclusion, I believe this article is index-able and have provided my subjective feedback for its improvement.

 Happiness!

 Gunnar Piho, PhD

Is the study design appropriate and does the work have academic merit?

Partly

Is the work clearly and accurately presented and does it cite the current literature?

Partly

If applicable, is the statistical analysis and its interpretation appropriate?

Not applicable

Are all the source data underlying the results available to ensure full reproducibility?

Partly

Are the conclusions drawn adequately supported by the results?

Partly

Are sufficient details of methods and analysis provided to allow replication by others?

Partly

Reviewer Expertise:

Health informatics, specializing in the primary and secondary use of health data, including data models, interoperability, privacy and transparency, and the architecture and software engineering of health information systems.

I confirm that I have read this submission and believe that I have an appropriate level of expertise to confirm that it is of an acceptable scientific standard, however I have significant reservations, as outlined above.

# Response

**Published:** 2024-08-17

**Authors:** Rada Hussein

**Affiliations:** Ludwig Boltzmann Institute for Digital Health and Prevention, Salzburg, Salzburg, Austria

Dear Prof. Gunnar Piho Thank you for your valuable review and constructive comments for improving the readability of the paper. We will consider all comments on the figures and tables in the second version of the paper. We will also provide more detailed description on the workshops Concerning the references, we directly refer to the technical specifications in the lessons learned column of the paper. Meanwhile, the provided the link of the project website in the first column of the table to refer to the project overview, deliverables and publication. However, we will check all references and revise them in the second version. In addition, we will enrich the results and discussion section with more elaboration on the project's objectives and the path to achieving these objectives as recommended. Appreciating your time and efforts, Rada
